# Case of Strangulated Hernia

**Published:** 1855-02

**Authors:** Samuel Thompson

**Affiliations:** Albion, Ill.


ART. II.—*Case of Strangulated Hernia. Operation and Recovery.* By SAMUEL THOMPSON, M.D., of Albion, Ill., delivered before the Æsculapian Society at their last meeting in Marshall, Ill.

Cases of Strangulated Hernia requiring the use of the knife are rather rare in country practice, and as the urgency of the symptoms often requires such prompt action, while the difficulty of procuring professional help or necessary conveniences at the time are so great, the rarity of such cases are very fortunate.

The subject of the following case, residing 10 miles from me, was a poor weakly emaciated woman of about 50 years of age, the mother of several children, she suffered for some years, part from a prolapsus of the uterus which protruded, even in the recumbent position, from the Vulva as a smooth round tumor, about as big as the end of a large egg. She stated she had been out of health for some time with chills and fever, and had been under the treatment of a quack Doctor, but had not had many chills since 1st Oct., 1853. I first saw her on 9th Oct. That after the chills left her she had some fever and colicky pains in her bowels, became costive, and discovered she had this tumor in the groin, (she had been washing and lifting an iron kettle) for this again she applied to her quack doctor, who told her "it was a rising" and directed her to poultice it, after continuing to do this for several days, she and her husband became alarmed, and sent for me (she had never noticed any rupture before this, but it might nevertheless have existed a long time). At this time there was a tumor about 2½ inches long, nearly egg shaped, with the apex towards the spine of the ilium, it occupied the left groin, and at its most prominent part, projected about ¾ inch above the surrounding integument. It was not tender on handling nor discolored; to the finger it felt firm and somewhat like an enlarged gland, there was no doughyness or elasticity, still in connection with the constipation, vomiting, hiccup &c, I felt satisfied that it was a hernia. I tried the taxis as long as under the circumstances I thought safe. And then considering that no purgatives had been tried, that she was free from pain, had no fever, passed her urine freely, while the tumor was not discolored or tender. I determined to try purgatives and enemata before resorting to the knife, I therefore with great care introduced about 18 inches of an œsophagus tube into the bowel, and then with an enema pump injected seven pints of warm water, holding in solution ʒiij Sulph. maj. This was mostly retained for about half an hour, when it was returned with a few small lumps of fecal matter. I left hydrg. sub-mur. gr. x. ext. hyos. gr. vi. in pills ij. to be given at 5½ P.M., followed in four hours by castor oil ℥i. spt. turpentine ʒi. and to be repeated every four hours, till three doses were taken, and directed that sinapisms be applied to the epigastrium to relieve the nausea.

The next day Oct 10th. I saw my patient about 11 A.M. Our enema and purgatives had been useless. She had not had any evacuation from the bowels. The nausea and vomiting with the hiccup, were continuous. The pulse was nearly imperceptible. The skin was cold; countenance bad; eyes dull and filmy, voice a mere whisper. It was evident there was no time to lose, and I regretted I had not operated on the previous visit, I however again tried the taxis for a few minutes, and considering the obscurity of the diagnosis, first directed my endeavours as for a femoral hernia, pressing downwords, supposing the gut had turned up over the edge of the femoral or saphenous opening after its escape. But I could not move it. I then for a minute endeavored to press it upwards and outwards in the direction of the inguinal canal, but with equal ill success. It was therefore evident that there was no time to lose in adopting the only means offering any chance of life, and even for that I feared there was hardly time, or strength to rally after the operation. I had sent to the two nearest houses, to get help of some kind, but none could be had, and therefore alone and unaided, except by the husband, an ignorant man, who had enough to do to support her head and apply stimulants and restoratives when needed. I had to proceed to the operation. The cabin was low with no window, and with a wide low porch over each door, I moved the bedstead across one of the doorways, laid my instruments on a reversed half bushel placed on a chair, having to pick out my own instruments, do my own sponging, &c., and not feeling quite certain whether the hernia was femoral or inguinal. I made an incision about 3 inches long, down the centre of the tumor, and carried merely through the skin, (cellular tissue there was none) and then commencing at the centre of the first incision, carried another downwards nearly at the right angles thus ⊤ and dissected up the flaps. The skin, and the different investing layers were very thin, and the latter dark colored, and closely adherent to one another. I had great difficulty in laying held of the sac, as there was no serum in it, and it was closely adherent to the bowels, though the adhesions were recent; but it had to be slowly separated, either with the director or the handle of the scalpel, upon opening the sac the bowel appeared tense dark colored, and full of fecal matter, and so dilated and flattened out, that it looked more like a mushroom or a button, the head of which was so large in proportion to the stem, that it was very difficult to get at the opening by which it had escaped, I drew the bowel slightly out, pushed in a finger, and finding the stricture to be at the superior falciform edge of the overlapping portion of the fascia lata femoris. I introduced a director and with a probe pointed bistoury directed upwards and slightly inwards towards the pubes, nicked edge of the fascia in two places, then slowly and easily emptied the tumor of its contents, the gut readily returned and having examined with my finger to ascertain that there was no further obstruction, I left her for a few minutes (laying down the flaps, &c) to recover herself, and to recover myself, for the fatigue of body and mind had been great, both from the constrained position and defective light in which I had to operate, and from the consciousness that any sudden movement on the part of my patient, might cause me to cut what could not be repaired, added to the doubt whether she would not die under my hands. I gave her a little spirits and water and laudanum, then brought the edges of the wound together with 3 points of interrupted suture, put on a compress soaked in oil, a roller round the body and crossing the perineum and thigh, allowed her to rest half an hour, and then administered an injection of warm water, which after being retained some time was returned with feculent matter of a dirty light brown color, her pulse became fuller, and having placed her in an easy position, I left her as follows: cal. gr. ix. quinine gr. vi. morph. sulph. gr. i. in cht. iij., one to be given every four hours. Light diet and perfect quiet.

Though working almost in the dark, no large vessel was cut, as I made constant reference to the indications of pulsation as recognized by my finger in the wound, not one ounce of blood was lost, indeed, had any hemorrhage occurred, I know not how I could have stopped it, having no assistance, and my patient was so low, that she would have sunk under a small loss —after removing the instruments &c., and exposing the hernia, it presented a singular appearance from a kind of second and external stricture produced over its apex or point by a nerve or a band of cellular matter, forming a very distinct depression or second strangulation.

It has been stated by Scarpa, Skesselbach, &c., that in crural hernia, there are symptoms which especially distinguish it, viz "a sense of stupor and heaviness in the thigh, œdema of the leg and even the foot of the same side," and the reason given, the pressure of the tumor upon the crural vessels and nerves appears good and sufficient. But in this case no such symptoms existed. But Scarpa further remarks, "that in women however, it is less easy to distinguish the crural hernia from the inguinal, and that the portion of the inferior pillar of the abdominal ring, which separates the opening from the internal and inferior angle of the crural arch is so slender in women, that it is sometimes hard to distinguish the crural from the inguinal hernia, which is not the case in male patients."

I saw my patient the next day, 11th, the bowels had been freely moved. she was indeed too much purged. But she had no fever, had eaten and slept, the wound looked healthy and healing, there was no tympanitis or abdominal tenderness.

Left opium gr. ij. cal. gr. ij. in chart to be given if too much purged.

12th. Patient looked much better, stools had been too frequent, but one of the powders had immediately checked them; she was cheerful, pulse natural, appetite good, tongue ditto. The edges of the wound seemed a little on the strain, I therefore clipped one of the stitches, the lips began to separate, and a sanious discharge escaped. I then closed the wound with collodion and cotton, leaving room for discharge. There was some slight tenderness about the umbilicus, she had had no stool that day. Calomel gr. v, morphine ½ gr. at night if uneasy. Oil in morning if bowels not moved.

14th. Mrs. R. doing well. No fever, appetite good, tongue clean, no pain or tenderness in abdomen, wound suppurating but gapes a little; starpped it up with adhesive plaster, and covered it with lint, spread with ungt. strammonii.

She perfectly recovered; but deid nine months after, with every symptom of introsusception. No post mortem obtained.

